# Ironically unwell: anaemia and iron deficiency among health-aware adults in the UK

**DOI:** 10.3389/fnut.2025.1679989

**Published:** 2025-10-07

**Authors:** Allister Irvine, Joanne Watt, Mary Jo Kurth, John V. Lamont, Le Roy Dowey, Peter Fitzgerald, Aaron Niblock, Alex Fairweather, Mark W. Ruddock

**Affiliations:** ^1^Randox Laboratories Ltd., Antrim, United Kingdom; ^2^School of Biomedical Sciences, Ulster University, Coleraine, United Kingdom; ^3^Department of Haematology, Antrim Area Hospital, Antrim, United Kingdom; ^4^Randox Health GB, London, United Kingdom

**Keywords:** anaemia, iron deficiency, iron supplementation, haemoglobin, ferritin, population study

## Abstract

**Background:**

Anaemia and iron deficiency are a global healthcare burden affecting almost 25% of the population. Many anaemia cases are caused by depletion of iron stores which can be treated by oral iron supplementation. However, anaemia may also result from functional iron deficiency, where chronic inflammation prevents utilisation of stored iron. Anaemia and iron deficiency are rarely profiled in general populations; however, they can have significant healthcare implications.

**Methods:**

Data from *n* = 33,029 serum samples were retrospectively analysed from individuals undertaking private health checks within Randox Health (UK). Samples were measured to detect anaemia, iron and vitamin deficiencies, based on established guidelines.

**Results:**

The overall prevalence of anaemia in the study was 6.0% (*n* = 1,917/31,803). The prevalence of anaemia was higher in females, with almost 1 in 10 (9.9%; *n* = 1,558/15,715) classified as anaemic; anaemia prevalence was highest in females aged 18–50 years. Similarly, absolute iron deficiency was also higher in females, with almost 1 in 3 (31.6%; *n* = 4,633/14,677) impacted. Functional iron deficiency was high in the study individuals across all age groups and sexes.

**Conclusion:**

The study identified that anaemia and iron deficiency are common underlying conditions in a health-conscious UK population. Despite the high prevalence of anaemia and iron deficiency burden on females of menstruating age, demonstrated in this study, and reported in the literature, screening for these conditions is not widespread. Should there be a national screening programme for anaemia and iron deficiency in females?

## 1 Introduction

Anaemia is a condition characterised by a reduced number of red blood cells (RBCs) or reduced haemoglobin concentration and is defined based on haemoglobin cut-offs (1). Anaemia has been identified as a significant healthcare problem, estimated to affect >1.92 billion individuals worldwide (2). Anaemia disproportionally impacts children and females with a worldwide prevalence of 41.4% in children <5 years and 31.2% in females, compared to 17.5% in males (2).

The World Health Organisation (WHO) has set a target to reduce the incidence of anaemia by 50% in females of reproductive age by 2030 (3). Despite this target, the incidence of anaemia has remained relatively unchanged over the last three decades (4). Worldwide, there are major disparities based on socioeconomic drivers, with low- and middle-income countries having a higher prevalence of anaemia, than high-income countries. For example, in 2021, Mali, Togo and Zambia reported that the prevalence of anaemia in their population was >50%, whereas Iceland, Norway and Monaco reported that prevalence of anaemia in their population was <5% (2).

While the causes of anaemia can be diverse and multifactorial, anaemia occurs through three main processes: (i) decreased production of RBCs, (ii) loss of blood and/or (iii) increased destruction of RBCs (5). Two thirds of anaemia cases worldwide are estimated to be caused by iron deficiency (2). Iron deficiency is the most common nutrient deficiency globally (6), and can occur without the development of anaemia; potential mechanisms include: (a) decreased iron intake, (b) decreased iron absorption, (c) increased iron demand and/or (d) chronic blood loss (7). Iron deficiency can be defined as absolute, where an individual’s iron stores are insufficient for their needs, or functional, where an individual has adequate iron stores, but iron supply for erythropoiesis is inadequate (8).

Iron availability is crucial for basic physiological processes, including DNA synthesis and repair, enzymatic activity, neurotransmission, and mitochondrial function (6). As a result, symptoms associated with iron deficiency vary widely and can include, but not limited to, fatigue, hair loss, weight gain, muscle and joint pain, and heart palpitations (9). The symptoms of iron deficiency and iron deficiency anaemia overlap considerably but are typically more severe in iron deficiency anaemia (9). Iron deficiency anaemia is also associated with reduced physical and cognitive performance in adults (7). During pregnancy, iron deficient anaemia is linked to low neonatal weight, increased risk of preterm labour, and the development of iron deficiency anaemia in infants (7).

Despite a lower prevalence of anaemia in high-income countries, there is a high burden associated with the treatment of iron deficient and anaemic individuals within healthcare systems. For example, the overall prevalence of anaemia in the Republic of Ireland healthcare system was 12.0%, and the condition was prevalent across all clinical departments (10). Additionally, in 2017/2018 in England, *n* = 97,781 and *n* = 159,400 patients were admitted to hospital with either a primary or secondary diagnosis of iron deficiency anaemia, respectively, at an estimated cost exceeding £90.6 million (11). Iron deficiency is also common in individuals with chronic inflammatory diseases, affecting 37–61% of patients with chronic heart failure, 24–85% of patients with chronic kidney disease, and 13–90% of patients with inflammatory bowel disease (7). Iron deficiency and anaemia are also important considerations in those undergoing surgery. For example, iron deficient patients undergoing cardiac or abdominal surgery have a higher risk of post operative fatigue and infection, and are more likely to require a blood transfusion (9). Additionally, post operative anaemia is associated with higher mortality and an increased length of hospital stay (7).

Multiple treatment options are available for both anaemia and iron deficiency. However, it is important that the underlying cause(s) of anaemia and/or iron deficiency are determined (7). For individuals with absolute iron deficiency, enhancement of dietary iron sources, or oral iron supplementation are typically the first choice of treatment to replenish iron stores (6). Various oral iron treatments are available; however, oral iron supplementation can be associated with unpleasant side effects including nausea, diarrhoea and constipation (6). Where oral iron supplementation is ineffective, or poorly tolerated, intravenous iron infusions can be administered (12). Intravenous administration of iron formulations is recommended for individuals where iron levels cannot be restored by oral iron supplementation (13). However, there are known side effects associated with intravenous iron administration, including chest tightness, joint pain and tachycardia (12). In extremely rare cases, more severe side effects can occur, including anaphylaxis, vomiting and loss of consciousness (12).

There is limited information on the prevalence of anaemia and iron deficiency in the general ‘healthy’ population. The aim of the current retrospective observational study was to determine the prevalence of anaemia and iron deficiency in a UK-based population undergoing private health checks.

## 2 Methods

### 2.1 Sample population

This observational study involved retrospective analysis of serum samples from *n* = 33,029 individuals who had visited a Randox Health Clinic within the UK for a health check between January 2022 and May 2024. Pregnant individuals were excluded from the study due to the differing serum ferritin and haemoglobin thresholds for anaemia and iron deficiency assessment during pregnancy. During an individual’s health check, measurements, including height (cm), weight (kg), hip circumference (cm), waist circumference (cm), pulse (beats per minute, bpm), systolic blood pressure (millimetre of mercury, mmHg), diastolic blood pressure (mmHg) and oxygen saturation (percent) were recorded by Randox Health clinic staff. Prior to, or during their health visit, individuals also completed a health and lifestyle questionnaire that allowed them to document health concerns or any symptoms they may be experiencing. Venous blood samples were collected and processed at the clinics and shipped to Randox Clinical Laboratory Services (RCLS) (Antrim, Northern Ireland, UK; ISO17025 accredited) for biomarker level determination. Informed consent from all study participants was recorded digitally through the Randox portal, and all individual data were anonymised. The study was reviewed and approved by Ulster University School of Biomedical Sciences Ethics Filter Committee (Project Number: FCBMS-25-073). All methods were carried out in accordance with the relevant guidelines and regulations and were conducted in accordance with the Declaration of Helsinki. It was not possible to involve patients or the public in the design, conduct, reporting, and dissemination plans of this research.

### 2.2 Sample biomarker analyses

Biomarker levels were determined by Randox Clinical Laboratory Services (RCLS) (Antrim, Northern Ireland, UK; ISO17025 accredited). Complete blood count indices (haemoglobin and RBC mean cell volume) were measured on a Sysmex XN-L 550 haematology analyser. Iron biomarkers (ferritin, iron, and total iron binding capacity) were measured using an Randox RX Imola clinical chemistry analyser. Vitamin biomarkers (vitamin B12 and folate) were measured using a Roche Cobas e801 immunology analyser. Transferrin saturation was calculated using the following equation:


$$\text{Transferrin Saturation}\;(\%)=\frac{\text{Serum iron}\;x\;100\;}{\text{Total}\;\mathrm{ir}on\;\text{binding capacity}}$$

Serum biomarker thresholds for determining deficiencies are shown in Table 1.

**Table 1:** Serum thresholds for determining anaemia, iron, and vitamin deficiencies.

Deficiency Type	Threshold
Anaemia (1)	Non pregnant female: haemoglobin <120 g/L Male: haemoglobin <130 g/L
Mild anaemia (1)	Non pregnant female: haemoglobin 110-119 g/L Male: haemoglobin 110- 129 g/L
Moderate anaemia (1)	Haemoglobin 80-109 g/L
Severe anaemia (1)	Haemoglobin <80 g/L
Microcytic anaemia (39)	Red blood cell mean cell volume <80 fl
Normocytic anaemia (39)	Red blood cell mean cell volume 80-100 fl
Macrocytic anaemia (39)	Red blood cell mean cell volume >100 fl
Absolute iron deficiency (22)	Serum ferritin <30 ng/mL
Functional iron deficiency (22)	Serum ferritin 30-99 ng/mL OR Serum ferritin 100-299 ng/mL and Transferrin saturation <20%
Vitamin B12 (40)	
B12 Deficiency	Serum B12 < 180 pg/mL
Possible B12 deficiency	Serum B12 180-350 pg/mL
Unlikely B12 deficiency	Serum B12 > 350 pg/mL
Folate (41)	
Folate deficiency	Serum folate <3 ng/mL
Possible folate deficiency	Serum folate 3-5 ng/ml
Unlikely folate deficiency	Serum folate >5 ng/ml

### 2.3 Statistical analyses

Analyses and graphs were generated using R, version 4.3.2 (https://www.R-project.org/; Vienna, Austria) (14). Continuous variables were statistically compared using Wilcoxon rank sum test and categorical variables were compared using Pearson’s Chi-squared test.

## 3 Results

Demographics for the study population are detailed in Table 2. From those who responded to the health questionnaire, the main reason for their visit(s) to the Randox Health Clinic was to assess general wellbeing (81.8%; *n* = 26,739/32,671). The remaining individuals indicated that their reason for attending the clinic was due to a health concern (18.2%; *n* = 5,932/32,671). Of those individuals who responded to the symptom questions, 59.9% (*n* = 17,296/28,876) reported experiencing no symptoms at the time of their clinic visit, and the remaining 40.1% (*n* = 11,580/28,876) reported experiencing symptoms.

**Table 2:** Demographics and biomarker results.

Characteristic	*n*	Female *n* = 16,660	Male *n* = 16,369^1^	*p*-value^2^
Age	33,029	41.2 (12.7)	42.8 (13.1)	<0.001
Ethnicity	32,648			<0.001
Asian		1,500 (9.1%)	1,425 (8.8%)	
Black		790 (4.8%)	543 (3.3%)	
Mixed		445 (2.7%)	346 (2.1%)	
Other		763 (4.7%)	608 (3.7%)	
White		12,905 (79%)	13,323 (82%)	
BMI	31,678	25.5 (5.7)	27.0 (4.4)	<0.001
Haemoglobin (g/L)	31,803	131.9 (10.5)	150.8 (10.7)	<0.001
Ferritin (μg/L)	30,666	64.4 (65.4)	177.4 (126.3)	<0.001
Transferrin Saturation (%)	29,164	27.9 (13.0)	33.2 (13.1)	<0.001
Vitamin B12 (ng/L)	29,269	550.9 (362.6)	525.7 (292.2)	0.013
Folate (μg/L)	28,819	10.8 (7.5)	9.1 (5.7)	<0.001

^1^Mean (SD); *n* (%).

^2^Wilcoxon rank sum test; Pearson’s Chi-squared test.

### 3.1 Anaemia prevalence

**Figure 1:**
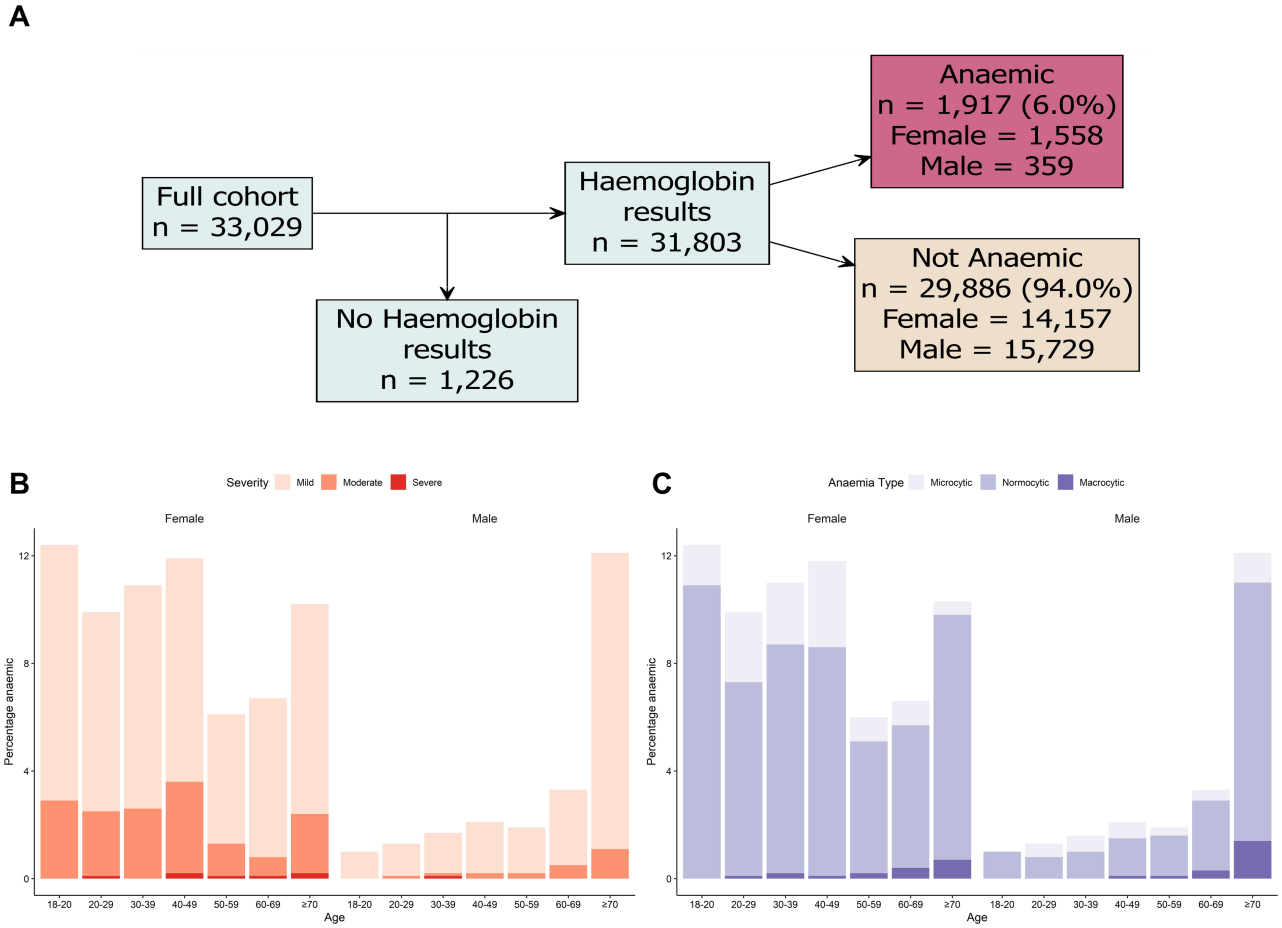
Prevalence of anaemia in study cohort. **(A)** Breakdown of haemoglobin results in the total study cohort. **(B)** Prevalence of anaemia severity by age and sex. **(C)** Prevalence of anaemia type based on age and sex. Biomarker cut-off used to determine iron and vitamin deficiencies are detailed in Table 1.

Anaemia severity was determined using haemoglobin thresholds as shown in Table 1. A total of 6.0% (*n* = 1,917/31,803) of individuals with haemoglobin results were classified as anaemic (Figure 1A). Anaemia was more common in females, (9.9%; *n* = 1,558/15,715) versus males (2.2%; *n* = 359/16,088). In females, the prevalence of anaemia was highest in individuals aged 18–49 years (>10% prevalence), and females ≥70 years (Figure 1B); a lower prevalence of anaemia was reported in females aged 50–69 years (<7% prevalence). When comparing individuals with anaemic results, the majority (77.8%; *n* = 1,492/1,917) were classified as mild anaemia, with 20.9% (*n* = 401/1,917) of results classified as moderate anaemia, and 1.3% (*n* = 24/1,917) classified as severe anaemia (Figure 1B). Anaemia results were classified as microcytic, normocytic and macrocytic, based on the RBC mean cell volume thresholds (Table 1). From the *n* = 1,917 anaemia results, 22.9% (*n* = 439/1,917) were classified as microcytic, 74.9% (*n* = 1,435/1,917) were classified as normocytic and 2.2% (*n* = 43/1,917) were classified as macrocytic (Figure 1C). Of the *n* = 1,721 anaemic individuals who answered symptom questions, the most common reported symptoms were fatigue (27.1%; *n* = 466/1,721), bloating (15.7%; *n* = 271/1,721), anxiety (13.1%; *n* = 225/1,721), low mood (13.1%; *n* = 225/1,721) and joint pain (13.0%; *n* = 224/1,721), while 57.1% (*n* = 982/1,721) of respondents reported no symptoms.

Of the individuals who responded on their health questionnaires, the majority (76.6%; *n* = 1,188/1,551) of anaemic females reported undertaking a health check to assess general wellbeing, rather than for a specific health concern (23.4%; *n* = 363/1,551). For the anaemic females who responded on the questionnaire, the majority reported they were menstruating total 79.1%; regular menstruation 62.9% (*n* = 887/1,411), irregular menstruation 16.2% (*n* = 229/1,411), and with fewer anaemic menopausal 4.6% (*n* = 65/1,411) and post-menopausal females (6.0%; *n* = 85/1,411). Additionally, heavy menstrual bleeding was common in anaemic females (45.5%; *n* = 509/1,118).

### 3.2 Iron deficiency prevalence

**Figure 2:**
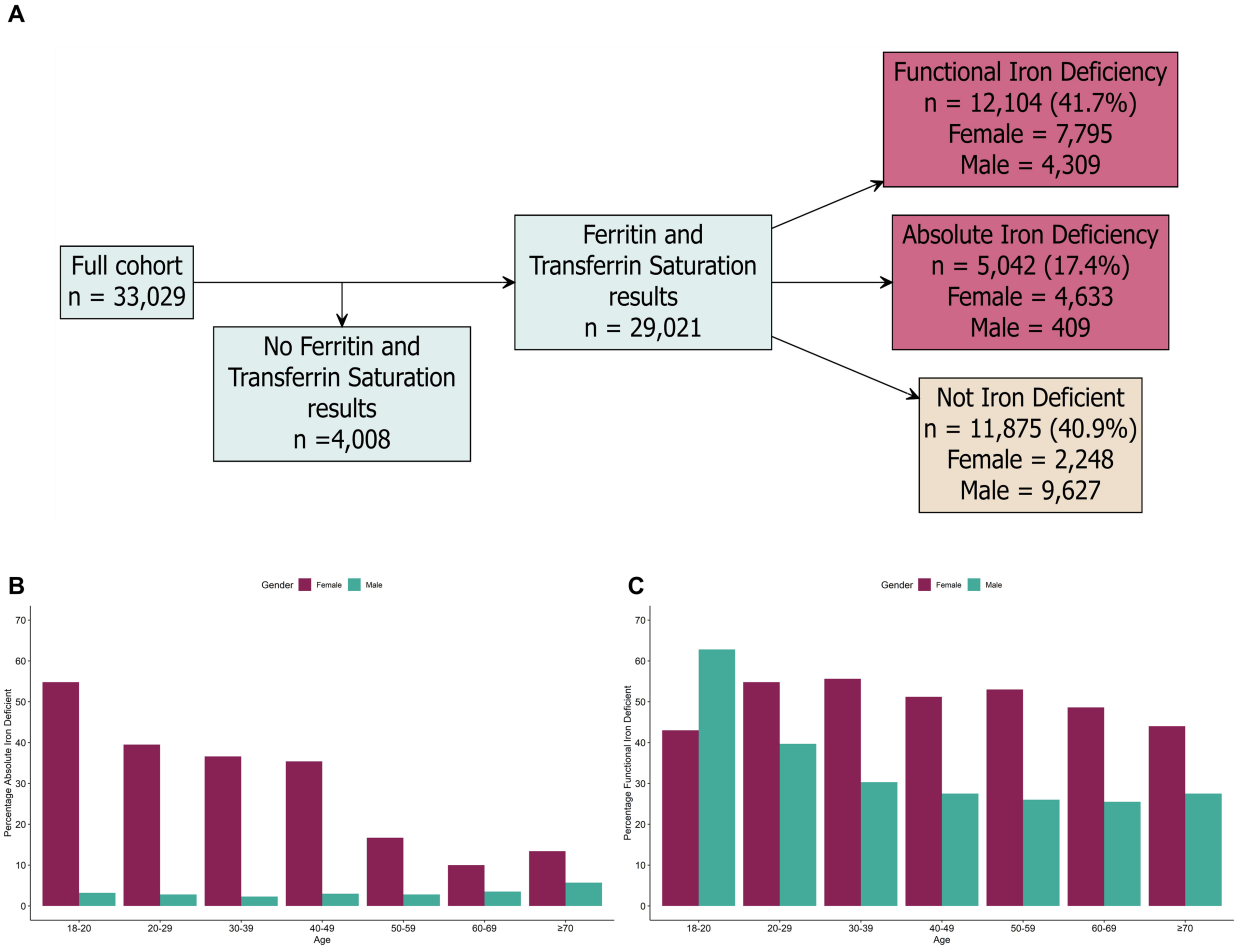
Prevalence of absolute and functional iron deficiency in the study cohort. **(A)** Breakdown of ferritin and transferrin saturation results in the total study cohort. **(B)** Prevalence of absolute iron deficiency by age and sex. **(C)** Prevalence of functional iron deficiency based on age and sex. Biomarker cut-offs used to determine iron and vitamin deficiencies are detailed in Table 1.

Absolute and functional iron deficiency were assessed using the ferritin and transferrin saturation criteria in Table 1. From the individuals with ferritin and transferrin saturation data, 17.4% (*n* = 5,042/29,021) had serum ferritin <30 ng/mL and were assigned as absolute iron deficient (Figure 2A). Absolute iron deficiency was more common in females compared to males 31.6% (*n* = 4,633/14,676) versus 2.9% (*n* = 409/14,346)). In females, absolute iron deficiency was high in females aged 18–49 years (>35% prevalence) and lower in females >50 years old (<20% prevalence) (Figure 2B). In contrast, absolute iron deficiency was low in all male age groups, with the highest prevalence in males >70 years (prevalence >5%) (Figure 2B). For individuals who completed the health questionnaire, the most common symptoms for absolute iron deficiency were fatigue (31.5%; *n* = 1,475/4,676), bloating (19.7%; *n* = 919/4,676), anxiety (16.9%; *n* = 791/4,676), low mood (16.1%; *n* = 753/4,676), and joint pain (13.6%; *n* = 638/4,676), while 52.4% (*n* = 2,450/4,676) reported no symptoms. In absolute iron deficient females who answered the questionnaire, the majority were of menstruating age (total 84.7%; regular menstruation 65.9% (*n* = 2,832/4,296), irregular menstruation 18.8% (*n* = 808/4,296), with lower numbers of menopausal (3.4%; *n* = 146/4,296), and post-menopausal (3.5%; *n* = 150/4,296) females. Heavy menstrual bleeding was also commonly reported for female individuals with absolute iron deficiency (47.5%; *n* = 1,735/3,652).

Functional iron deficiency was high in the study population; 41.7% (*n* = 12,104/29,021) of individuals with ferritin and transferrin saturation information were assigned as functional iron deficiency (Figure 2A). Functional iron deficiency was more common in females (53.1% (*n* = 7,795/14,676) of females), compared to males (30.0% (*n* = 4,309/14,345) of males). Functional iron deficiency was high (>40% prevalence) in all female age groups; for males, the functional iron deficiency was highest in 18-20-year-olds (>65% prevalence) (Figure 2C). For individuals with functional iron deficiency that completed the health questionnaire, the most common symptoms included fatigue (27.0%; *n* = 2,991/11,067), bloating (15.3%; *n* = 1,689/11,067), anxiety (14.9%; *n* = 1,648/11,067), low mood (13.3%; *n* = 1,475/11,067), and joint pain (12.8%; *n* = 1,420/11,067); while 56.6% (*n* = 6,268/11,067) reported no symptoms.

### 3.3 Vitamin deficiency prevalence

**Figure 3:**
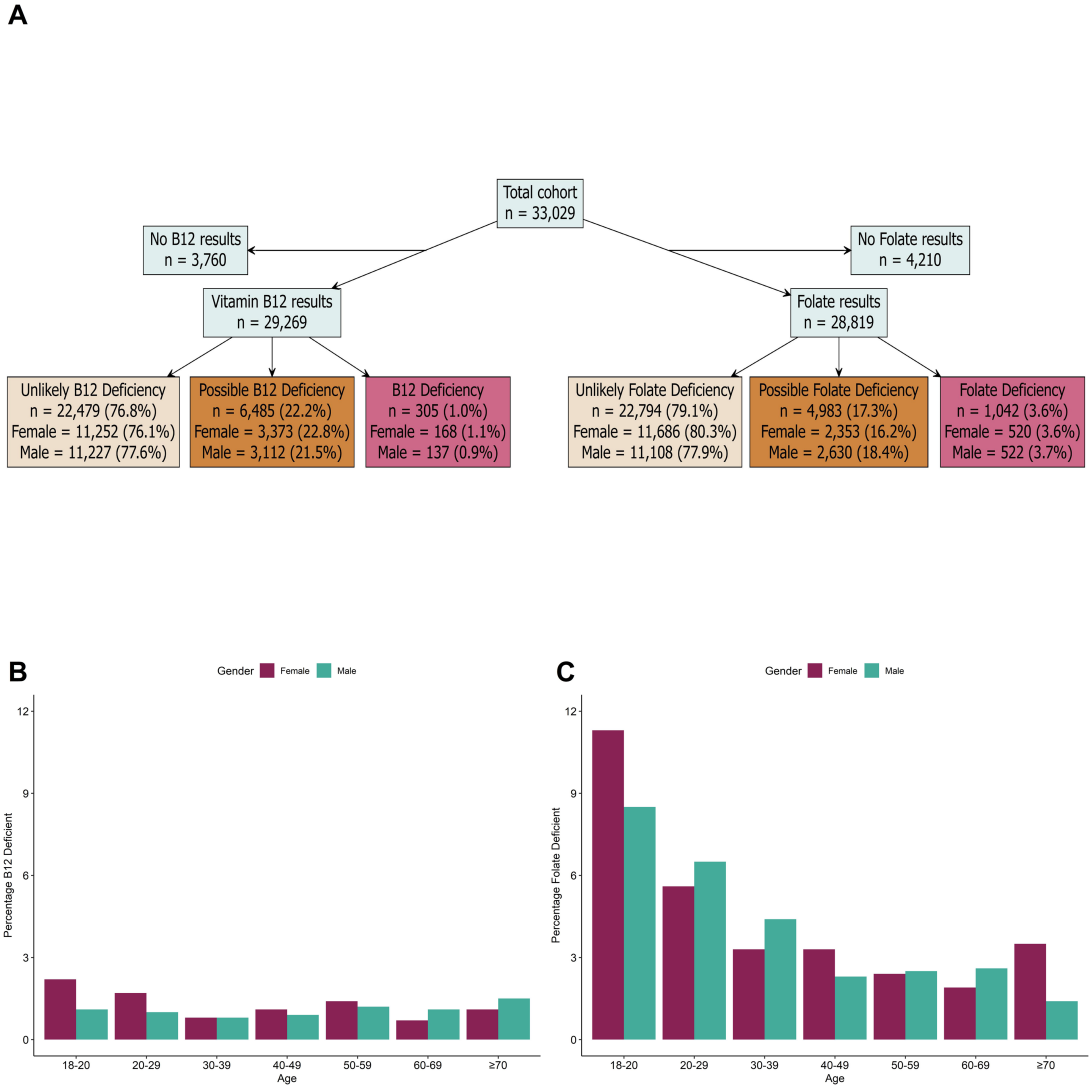
Prevalence of vitamin deficiencies in the study cohort. **(A)** Breakdown of B12 and folate results in the total study cohort. **(B)** Prevalence of vitamin B12 deficiency by age and sex. **(C)** Prevalence of folate deficiency based on age and sex. Biomarker cut-offs to determine iron and vitamin deficiencies are detailed in Table 1.

Folate and vitamin B12 deficiencies were defined according to the serum thresholds described in Table 1. From the individuals with vitamin B12 data, 1.0% (*n* = 305/29,269) were defined as B12 deficient and 22.2% (6,485/29,269) were defined as possible B12 deficiency (Figure 3A). The overall level of vitamin B12 deficiency was largely consistent across all age groups and sex (Figure 3B). For individuals who completed the health questionnaire, the most common symptoms for vitamin B12 deficiency were fatigue (24.7%; *n* = 67/271), bloating (14.4%; *n* = 39/271), low mood (12.5%; *n* = 34/271), anxiety (11.8%; *n* = 32/271) and anxiety (11.4%; *n* = 31/271); while 60.9% (*n* = 165/271) of individuals reported no symptoms. From the individuals with folate data, 3.6% (1,042/28,819) were defined as folate deficiency and 17.3% (4,983/28,819) were defined as possible folate deficiency (Figure 3A). Folate deficiency was highest (>5% prevalence) in males and females <30 years old and levels were largely consistent amongst older age groups (~3% prevalence in >30s; Figure 3C). Similarly, the most common symptoms for folate deficiency included fatigue (31.4%; *n* = 299/953), anxiety (16.1%; *n* = 153/953), low mood (15.2%; *n* = 145/953), bloating (15.2%; *n* = 145/953), and joint pain (14.4%; *n* = 137/953); while 55.5% (*n* = 529/953) of individuals reported no symptoms.

### 3.4 Potential causes of anaemia

**Figure 4:**
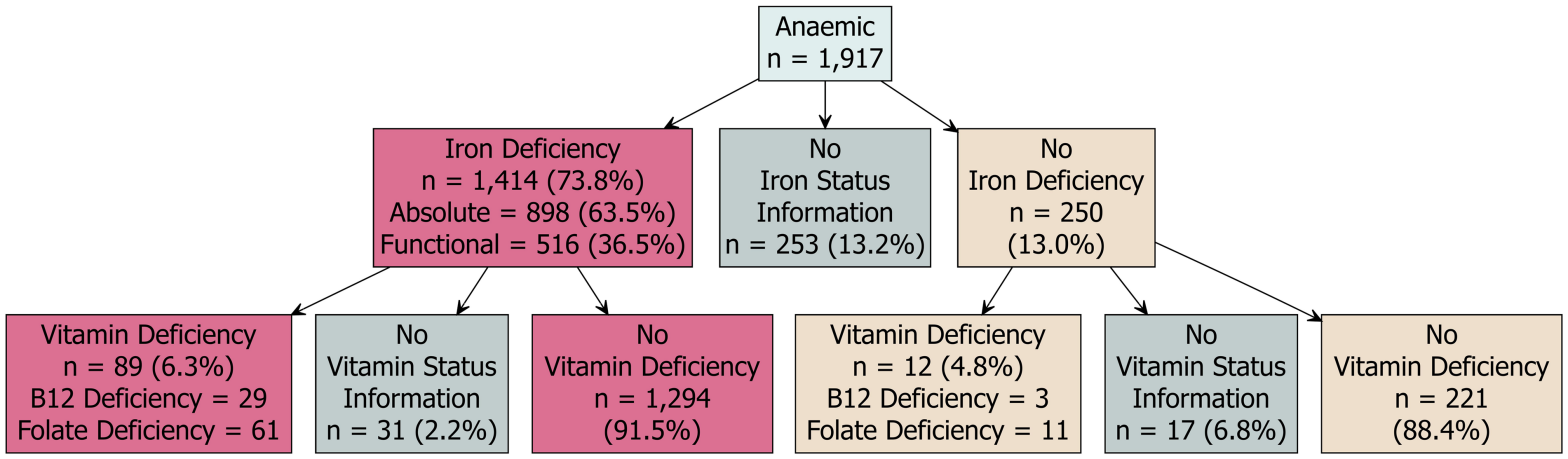
Breakdown of iron and vitamin deficiencies in the anaemic cohort. Biomarker cut-offs to determine iron and vitamin deficiencies are detailed in Table 1.

To investigate potential causes of anaemia in the study cohort we explored the prevalence of iron and vitamin deficiencies among the anaemic results. Iron and vitamin status results were not available for 13.2% (*n* = 253/1,917) and 13.0% (*n* = 249/1,917) anaemic individuals, respectively. Analysis of the anaemic results indicated that the majority were also iron deficient (73.8%; *n* = 1,414/1,917) (Figure 4). Of these individuals, 63.5% (*n* = 898/1,414) had absolute iron deficiency, and 36.5% (*n* = 516/1,414) had functional iron deficiency. A small proportion of anaemic individuals with some form of iron deficiency also had vitamin deficiencies (6.3%; *n* = 89/1,414) (Figure 4). Of those individuals with anaemia, 13.0% (*n* = 250/1,917) had results indicating they were not iron deficient. However, a small proportion (4.8%; *n* = 12/250) had vitamin deficiencies. Vitamin status information was not available for 6.8% (*n* = 17/250) of individuals and the remaining 88.4% (*n* = 221/250) were not vitamin deficient.

## 4 Discussion

In this retrospective observational study, the prevalence of anaemia and iron deficiency were investigated in a cohort of apparently healthy individuals (*n* = 33,029) from the UK who were proactive in assessing their health through private health checks. Interestingly, 59.9% of the individuals included in this study, reported that they had no symptoms and 81.8% were attending for a health check to monitor general wellbeing rather than for a specific health concern.

In the individuals who attended for a private health check, the overall prevalence of anaemia was 6.0%, which is similar to the prevalence observed for the UK population (6.5%) in the 2021 Global Burden of Disease study (2). The WHO defines prevalence of anaemia higher than 4.9% as a finding of mild public health significance (1). In the current observational study, the prevalence of anaemia was higher in females (10%) when compared to males (<3%). These findings are consistent with WHO estimates on anaemia prevalence in females aged 15–49 in the UK of 10.9% (15).

Most anaemia cases in this study were defined as mild (77.8%), with few individuals suffering from severe anaemia (1.3%). Of the individuals who recorded symptoms on their health questionnaires, the most prevalent symptoms in anaemic individuals were fatigue, joint pain and headaches. However, even mild anaemia is associated with higher postoperative mortality and morbidity in patients undergoing major non-cardiac surgery compared to patients who are not anaemic (16).

In this study, a small proportion of individuals (11.5%) who were anaemic, had no iron or vitamin deficiencies, however, 73.8% of individuals with anaemia, also had either absolute or functional iron deficiency, indicating that the iron deficiency was the most likely cause for their anaemia. A small proportion of individuals (4.8%) who were not iron deficient, had vitamin B12 or folate deficiencies, which may have contributed to their anaemia. Thus, it is important to investigate the cause of the deficiency to enable appropriate treatment and management to alleviate symptoms.

It is estimated that one third of iron deficiency anaemia cases in males and post-menopausal females are caused by underlying pathological abnormalities, including gastrointestinal tract irregularities (8). The British Society of Gastroenterology recommends urgent referrals for gastrointestinal investigation in adults with unexplained iron deficiency anaemia (8). Furthermore, anaemia can also be caused by autoimmune conditions (17) or inherited conditions such as thalassemia (18).

Absolute and functional iron deficiency are rarely reported in generally healthy populations. We observed a high prevalence of iron deficiency in the study cohort with 84.7% of females and 32.9% of males having either absolute, or functional iron deficiency. Almost a third (31.6%) of females had serum ferritin levels indicating absolute iron deficiency compared to only 2.9% of males. In females aged 18–49 years, the prevalence of absolute iron deficiency exceeded 35%; iron deficiency was more common in females who reported regular menstruation and/or those who experience heavy menstrual bleeding. These figures reflect results reported in studies from UK, US and Canada. In two UK anaemia and iron deficiency screening trials, 35% of female nurses and 48% of female runners were reported to be iron deficient (serum ferritin cut-off of <30 μg/L) (19). In a US and Canadian-based study, a high prevalence of absolute iron deficiency of >21% (based on a more stringent serum ferritin cut-off of 25 ng/mL) in females aged 25–54 years was reported (20). Similarly, in a large population study based in Canada, 38.3% of females aged 15–54 years were classified as iron deficient (serum ferritin cut-off of <30 μg/L) (21).

A high proportion of results from individuals in this study indicated they had functional iron deficiency. While functional iron deficiency is typically not quantified in general populations, recent studies have highlighted that the prevalence of functional iron deficiency is high in a general population (22). In a German longitudinal health study involving 5,000 participants, 54.5% of the participants were functionally iron deficient during an initial health visit (22). Significantly, both functional and absolute iron deficiency were associated with a 30 and 90% increase in all-cause mortality, respectively (22). Functional iron deficiency is commonly associated with individuals with inflammatory conditions including infections, obesity, cancer, and chronic kidney disease (13).

One of the major risk factors for the development of anaemia and/or iron deficiency in females of menstruating age is heavy menstrual bleeding (23). There are major differences in the estimated prevalence of heavy menstrual bleeding amongst general populations with ranges from 3 to 50% depending on the definitions applied (23). In the anaemic females in this study, the overall prevalence of self-reported heavy menstrual bleeding was high and may be contributing to their anaemic status. It is important to note, females may be unaware that they suffer from heavy menstrual bleeding, due to a lack of awareness of what constitutes unusual menstrual bleeding, and the normalisation of symptoms (23). Current guidelines on the consideration of anaemia and iron deficiency in females with heavy menstrual bleeding are insufficient and contradictory (23).

Despite the widespread understanding that menstruating females are at higher risk of iron deficiency and anaemia and various studies reporting high iron deficiency and anaemia prevalence, testing for these conditions in healthy females is not widespread (5). Moreover, even regular health checks for anaemia and iron deficiency in high-risk groups such as pregnant females are inadequate (24). For example, in Saudi Arabia, where haemoglobin and ferritin testing are recommended at every trimester, only 51.5% of expected haemoglobin tests were conducted, and <5% of pregnant females were tested for ferritin (25). Recently, the International Federation of Gynaecology and Obstetrics recommended that all females of reproductive age, should be tested for iron deficiency (26). However, these recommendations have not been adopted in healthcare settings. Surprisingly, in this current study, in individuals who were anaemic, or were absolute iron deficient, 57.1 and 52.4%, respectively, reported no symptoms. Without the individuals undergoing private health tests, their anaemia and/or iron deficiency may have been unreported. These data support the importance of testing healthy populations in identifying hidden anaemia and iron deficiency.

The current study demonstrated a high prevalence of iron deficiency and anaemia amongst the population. The UK has a higher estimated years living with disability attributed to anaemia (105.5 per 100,000 of the population) compared to Western Europe (average of 89.0 per 100,000 of the population) (2). Notably, these estimates do not consider individuals with iron deficiency without anaemia, and the associated disability burden. Various studies have indicated that anaemia negatively impacts worker productivity, contributing to absenteeism, in both labour and non-labour-intensive occupations (27). In addition, iron deficiency has been linked to symptoms associated with depression (28, 29) as well as being a risk factor for the development of postpartum depression (30). Additionally, iron dysregulation and inflammatory anaemia has been identified as a contributor to the development of post-acute sequelae of Coronavirus Disease 2019 (also known as ‘long covid’) (31).

While the levels of haemoglobin used in this study to define anaemia are based on recently updated WHO guidelines, alternative levels of haemoglobin have been suggested (5). Similarly, different ferritin thresholds have been suggested for defining iron deficiency (13, 20). While the gold standard for an absolute iron deficiency diagnosis relies on iron staining in bone marrow, bone marrow aspiration is an invasive procedure that is rarely conducted in cases where iron deficiency is suspected (6). Recently a study suggested that a transferrin saturation cut-off of <20%, rather than ferritin-based metrics, in patients with chronic heart failure, might be more useful to define iron deficiency, and predict whether intravenous administration of iron could be beneficial (32). It is important to note that ferritin levels can be impacted by inflammation and other factors (9). Recently WHO guidelines have recommended the use of other inflammatory markers including C-reactive protein and *α*-1 acid glycoprotein (AGP) when assessing serum ferritin in areas of widespread infection or inflammation (1).

It is important to acknowledge that the cohort in this study is unlikely to be fully representative of the UK population. Participants choosing to access private healthcare services potentially have a higher educational level, and income, and are less likely to experience economic hardship or nutritional deficiencies related to poverty. Given this context, the high prevalence of anaemia and iron deficiency observed in this group is concerning and may suggest even higher rates in economically disadvantaged communities. Furthermore, while the cohort was described as generally healthy, 18% were classified as obese and 35% as overweight based on NHS BMI criteria (33). Interestingly, these figures are similar to national statistics for England, where 26% of adults are obese and 38% are overweight (34).

## 5 Conclusion

Anaemia and iron deficiency are among the most preventable and treatable health conditions—yet routine health checks remain uncommon. This study revealed a high prevalence of both conditions within a health-conscious UK population undergoing private health assessments. Despite their proactive approach to health, one in ten females in this cohort were anaemic, and one in three had absolute iron deficiency. These findings are particularly troubling given that this group is unlikely to face the socioeconomic barriers that typically exacerbate nutritional deficiencies. Moreover, the study uncovered a widespread presence of functional iron deficiency across all sexes and age groups, highlighting an overlooked issue that requires further investigation into its underlying causes and clinical consequences. Considering the well-documented risks of untreated iron deficiency, including fatigue, cognitive impairment, and reduced quality of life, it is concerning that systematic testing, especially for females, is not standard practice. This study raises the question for population-based screening of anaemia and iron deficiency in high-risk groups.

## 6 Limitations of the study

There were limitations associated with this study; access to full information on the medication history of the individuals involved was limited. Various medications can impact the absorption of iron (e.g., proton pump inhibitors) (35) or can cause gastrointestinal bleeding events (e.g., non-steroidal anti-inflammatory drugs) (36) and may have influenced the prevalence of iron deficiency and anaemia observed in this study. Additionally, some of the individuals included in this study may have already been diagnosed as iron deficient and/or anaemic and may have already started iron treatments.

The majority of the population included in this study were Caucasian (79.4%). Ethnicity is a well-known risk factor which can influence the prevalence of anaemia and nutritional deficiencies. Additionally, BMI is also known to impact anaemia and nutritional deficiencies. We have included information on ethnicity and BMI across the anaemia and nutritional deficiency cohorts in Supplementary Table 1, and further investigations into these factors are warranted in a larger population study.

Ferritin and transferrin are acute phase proteins, and levels can be affected by infection, inflammation, dietary intake and contraceptive methods (e.g., copper intrauterine device) (37). In addition, chronic disease caused by conditions such as chronic kidney disease and auto-immune disease are a major cause of anaemia and iron deficiency (38). Information on an individual’s co-morbidities was limited, including whether they had a history of diseases associated with anaemia or nutritional deficiencies. Therefore, information on chronic disease or inflammation causing conditions would have been beneficial in considering iron and anaemia status.

## Data Availability

The raw data supporting the conclusions of this article will be made available by the authors, without undue reservation.
