# Supplementary material for: Ironically unwell: anaemia and iron deficiency among health-aware adults in the UK
Source: Front Nutr. 2025 Oct 7;12:1679989. doi: 10.3389/fnut.2025.1679989 (PMC12537364; doi:10.3389/fnut.2025.1679989)
Supplement: Supplementary file 1 [file Table_1.DOCX]

**Supplementary Table 1: Breakdown of anaemia and nutritional deficiency cohorts by body mass index (BMI) group and ethnicity**

| **Characteristic** | **Full Cohort**  n =33,029*^1^* | **Anaemic**  n = 1,917*^1^* | **Absolute Iron Deficient**  n =5,042*^1^* | **Functional Iron Deficient**  n =12,104*^1^* | **B12 Deficient** n = 305*^1^* | **Folate Deficient**  n = 1,042*^1^* | **p-value***^2^* |
| --- | --- | --- | --- | --- | --- | --- | --- |
| **BMI Group** |  |  |  |  |  |  | <0.001 |
| Underweight  (BMI <18.5) | 675 (2.1%) | 72 (3.9%) | 185 (3.8%) | 321 (2.7%) | 2 (0.7%) | 22 (2.2%) |  |
| Healthy weight  (BMI 18.5-24.9) | 13,853 (44%) | 998 (54%) | 2,634 (54%) | 5,695 (48%) | 115 (39%) | 298 (29%) |  |
| Overweight  (BMI 25-29.9) | 11,026 (35%) | 455 (25%) | 1,211 (25%) | 3,691 (31%) | 107 (36%) | 331 (32%) |  |
| Obese  (BMI >30) | 6,124 (19%) | 326 (18%) | 839 (17%) | 2,048 (17%) | 73 (25%) | 368 (36%) |  |
| NA | 1,351 | 66 | 173 | 349 | 8 | 23 |  |
| **Ethnicity** |  |  |  |  |  |  | <0.001 |
| Asian | 2,925 (9.0%) | 308 (16%) | 575 (12%) | 1,122 (9.4%) | 63 (21%) | 87 (8.4%) |  |
| Black | 1,333 (4.1%) | 275 (14%) | 307 (6.2%) | 452 (3.8%) | 7 (2.3%) | 63 (6.1%) |  |
| Mixed | 791 (2.4%) | 65 (3.4%) | 148 (3.0%) | 328 (2.7%) | 4 (1.3%) | 17 (1.6%) |  |
| Other | 1,371 (4.2%) | 140 (7.3%) | 263 (5.3%) | 518 (4.3%) | 20 (6.6%) | 36 (3.5%) |  |
| White | 26,228 (80%) | 1,121 (59%) | 3,667 (74%) | 9,529 (80%) | 210 (69%) | 832 (80%) |  |
| NA | 381 | 8 | 82 | 155 | 1 | 7 |  |

*^1^*n (%)

*^2^*Pearson's Chi-squared test
